# Prevalencia de *Chlamydia trachomatis* en la población femenina asintomática atendida en los servicios de citología cervical de tres instituciones prestadoras de servicios de salud en Medellín, Colombia

**DOI:** 10.7705/biomedica.5225

**Published:** 2020-06-30

**Authors:** Nataly Orozco-Hoyos, Armando Baena, Carolina Montoya-Ruiz, Gloria I. Sánchez, Eliana Restrepo

**Affiliations:** 1 Grupo de Bacterias y Cáncer, Universidad de Antioquia, Medellín, Colombia Universidad de Antioquia Grupo de Bacterias y Cáncer Universidad de Antioquia Medellín Colombia; 2 Grupo de Infección y Cáncer, Universidad de Antioquia, Medellín, Colombia Universidad de Antioquia Grupo de Infección y Cáncer Universidad de Antioquia Medellín Colombia; 3 Laboratorio de Diagnóstico Molecular y Bioinformática, Universidad de los Andes, Bogotá D.C, Colombia Universidad de los Andes Universidad de los Andes BogotáD.C Colombia

**Keywords:** Chlamydia trachomatis, enfermedades de transmisión sexual, prevalencia, factores de riesgo, infecciones por papilomavirus, Colombia, Chlamydia trachomatis, sexually transmitted diseases, prevalence, risk factors, papillomavirus infections, Colombia

## Abstract

**Introducción.:**

La infección de transmisión sexual causada por *Chlamydia trachomatis* es la más frecuente de etiología bacteriana en el mundo. Aunque puede ser asintomática en la mayoría de los casos, *C. trachomatis* puede generar diversos problemas de salud reproductiva en mujeres, como cervicitis, endometritis y salpingitis. A pesar de su importancia, en Medellín no se cuenta con suficientes datos epidemiológicos sobre esta infección.

**Objetivo.:**

Determinar la prevalencia de *C. trachomatis* en mujeres de Medellín, determinar los posibles factores de riesgo y evaluar la relación con la infección por el virus de papiloma humano (HPV).

**Materiales y métodos.:**

Se hizo un estudio transversal multicéntrico para detectar la infección por *C. trachomatis* en 1.282 mujeres mediante reacción en cadena de la polimerasa (PCR) convencional y el estuche comercial LightMix 480 HT CT/NG™ (Roche, Basilea, Suiza).

**Resultados.:**

La prevalencia total de la infección por *C. trachomatis* fue de 4,1 % (IC_95%_ 2,9-5,3). Se encontró una relación significativa de la infección con la edad, el consumo de cigarrillo y el uso de anticonceptivos hormonales.

**Conclusión.:**

La prevalencia de la infección es similar a la reportada en otros lugares del país y del mundo, siendo las mujeres más jóvenes las más afectadas. En cuanto a la presentación del HPV, no se encontró ningún tipo de relación con *C. trachomatis*.

## Abstract (en)

**Introduction:**

The sexually transmitted infection caused by *Chlamydia trachomatis* is the most common of bacterial etiology in the world. Although it can be asymptomatic in most cases, it can produce several reproductive health problems in women such as cervicitis, endometritis, and salpingitis. Despite its importance, the epidemiological data on this infection is insufficient in Medellín.

**Objective:**

To determine the prevalence of *C. trachomatis* in women from Medellín, identify possible risk factors, and evaluate its relationship with the human papillomavirus infection (HPV).

**Materials and methods:**

We conducted a multicenter cross-sectional study to detect *C. trachomatis* infection in 1,282 women using PCR and the LightMix 480 HT CT/NG™ commercial kit (Roche, Basel, Switzerland).

**Results:**

The total prevalence of *C. trachomatis* infection was 4.1% (95% CI: 2.9-5.3). We found an association between the presence of the infection and age, cigarette consumption, and the use of hormonal contraceptives.

**Conclusion:**

The prevalence of the infection is similar to that reported in other parts of the world with young women being the most affected population. No relation was found with the presence of HPV.

*Chlamydia trachomatis* es el agente causal de la infección de transmisión sexual de origen bacteriano más frecuente en el mundo. Se estima que ocurren 131 millones de casos nuevos cada año ^1^. Esta infección se transmite durante las relaciones sexuales vaginales, orales o anales, y de madre a hijo durante el parto vaginal. En el 70 % de los casos en mujeres y en el 50 % en hombres, la infección transcurre de manera asintomática y permanece clínicamente indetectable ^2^.

En la mujer, *C. trachomatis* puede producir cervicitis, endometritis y salpingitis, y dejar secuelas a largo plazo como enfermedad inflamatoria pélvica, embarazo ectópico e infertilidad. La duración de la infección genital no tratada y sin complicaciones es aproximadamente de un año, con una tasa de resolución espontánea de 11 a 44 %. Sin embargo, el 40 % de las mujeres con infección por C. *trachomatis* no tratadas desarrollan enfermedad inflamatoria pélvica y, de ellas, el 20 % desarrolla infertilidad, el 18 % experimenta dolor debilitante crónico en la pelvis, y el 9 % presenta embarazos ectópicos ^2^.

Debido a la gran proporción de pacientes asintomáticas y a las graves secuelas de la infección, la vigilancia epidemiológica es importante. No obstante, en Colombia la información epidemiológica es limitada y son pocos los estudios que han explorado los factores de riesgo para dicha infección. En un estudio de 1.990 en mujeres asintomáticas de Bogotá, se detectó mediante métodos microbiológicos una prevalencia de *C. trachomatis* del 25 % en mujeres que asistían a consulta ginecológica regular ^3^. En otro estudio más reciente entre estudiantes de secundaria de la misma ciudad, se tomaron muestras de orina de hombres y mujeres sexualmente activos y se detectó mediante métodos moleculares una prevalencia de 2,24 % ^4^. En la misma ciudad, Cardona- Arias, *et al.,* encontraron 15,6 % de mujeres asintomáticas seropositivas para anticuerpos IgG ^5^, en tanto que, en el 9,7 % de las mujeres con infección vaginal de la misma ciudad, se encontró el genoma de *C. trachomatis*, lo cual resalta la importancia de este agente en la población colombiana ^6^.

A pesar de su importancia, en Medellín apenas se han realizado dos estudios de vigilancia. En el primero, de Robledo, *et al.* (1987), se evaluó la presencia de esta infección en mujeres con cervicitis y se encontró una positividad del 20,0 % ^7^*.* En el segundo, de Cardona-Arias, *et al.* (2012), se encontró que la presencia de anticuerpos IgG en mujeres con factores de riesgo para infección de transmisión sexual fue de 16,9 % ^5^*.* Estos datos sugieren que existe una proporción importante de mujeres asintomáticas infectadas con *C. trachomatis* en riesgo de desarrollar posibles complicaciones en caso de no recibir tratamiento.

Por otra parte, se sabe poco sobre la relación epidemiológica de esta infección con otras de transmisión sexual muy frecuentes en el país, como la causada por el virus del papiloma humano (HPV). El estudio de dicha asociación es de gran importancia, ya que, según información previa, la infección persistente de *C. trachomatis* genera inflamación crónica y, eventualmente, metaplasia, por lo cual se considera un factor de riesgo para el desarrollo de cáncer de cuello uterino ^8^^,^^9^. En estudios anteriores, se detectó *C. trachomatis* mediante técnicas moleculares en una cohorte de 219 pacientes de Bogotá y Girardot (Cundinamarca, Colombia) y de Chaparral (Tolima, Colombia) infectadas con HPV, y se estableció una frecuencia de la infección del 28 %. Se observó, además, que las mujeres positivas para *C. trachomatis* generalmente estaban infectadas con múltiples genotipos del HPV ^10^

A pesar de la importancia de este agente, se tiene poca información sobre los posibles factores de riesgo y las conductas sexuales que favorecerían la presencia de la infección. En este contexto, en el presente estudio se buscó determinar la presencia de la infección por *C. trachomatis* en mujeres asintomáticas de Medellín, su prevalencia y los factores de riesgo asociados, así como evaluar su asociación con la infección por HPV.

## Materiales y métodos

### Diseño del estudio y recolección de los datos sociodemográficos

Se hizo un estudio descriptivo de corte transversal que incluyó 1.282 mujeres participantes en el estudio “Papel de la variabilidad molecular de la proteína E6 del VPH16 y su relación con el HLA en el desarrollo de cáncer cervical en Colombia” (Grupo de Infección y Cáncer, Universidad de Antioquia), a quienes se les tomaron muestras de exudado cervical. Las mujeres fueron atendidas entre el 2008 y el 2010 en los servicios de citología de tres instituciones prestadoras de servicios de salud (IPS) de Medellín: el Laboratorio de la Sede Ambulatoria de la IPS Universitaria (Universidad de Antioquia), el Laboratorio de Dinámica IPS, sede calle 33, y el Laboratorio de Dinámica IPS, sede centro ^11^. En el marco del proyecto original se había extraído ADN de las muestras cervicales y se habían tipificado para detectar el HPV. Las participantes habían dado su consentimiento para el uso de las muestras cervicales en futuros estudios.

Se excluyeron las mujeres en embarazo, aquellas con lesión intraepitelial de alto grado (LIE-AG), las positivas para el virus de inmunodeficiencia humana (HIV), las sometidas previamente a histerectomía, conización, radioterapia o quimioterapia, así como aquellas con incompetencia mental. Se excluyeron también las mujeres menores de 20 años, aquellas con muestra de ADN cervical negativa para β-globina o con insuficiente ADN para practicar las pruebas. A las participantes se les solicitaron sus datos sociodemográficos e información sobre su comportamiento sexual y reproductivo, factores infecciosos, resultados de citología y consumo de cigarrillo.

### Toma de muestras y diagnóstico de C. trachomatis

A todas las participantes se les practicó una citología convencional y se les tomó una muestra de exudado cervical con un citocepillo que, posteriormente, se introdujo en un tubo con medio de transporte (PreservCyt^™^, Cytyc Corporation, Boxborough, Estados Unidos) y se llevó al laboratorio del Grupo de Infección y Cáncer donde se congeló a -20 °C hasta su procesamiento.

En cada una de las muestras, se extrajo ADN mediante la técnica de proteinasa K (PK) (Sigma-Aldrich, St. Louis, Estados Unidos) siguiendo las recomendaciones del fabricante. El ADN extraído se cuantificó usando un espectrofotómetro NanoDrop 2000c^™^ (Thermo Scientific, Estados Unidos), cuya calidad fue evaluada mediante PCR amplificando un fragmento de 209 pb del gen de la β-globina utilizando el protocolo previamente descrito ^12^.

A partir de las muestras positivas para β-globina, se detectó *C. trachomatis* mediante PCR convencional amplificando un fragmento de 495 pb correspondiente al plásmido críptico y usando los cebadores descritos por Petrovay, *et al.*, y la enzima ADN Polimerasa *Taq*™ (Thermo Scientific, Massachusetts, Estados Unidos) ^13^. Los productos de la amplificación se observaron mediante electroforesis en gel de agarosa teñido con bromuro de etidio. Para confirmar la reproducibilidad de la PCR convencional de *C. trachomatis*, todas las muestras positivas se evaluaron por duplicado.

Además, todas las muestras positivas y el 10 % de las negativas, elegidas al azar, se evaluaron también usando el sistema LightMix 480 HT CT/NG™ (Roche, Basilea, Suiza). Este método amplifica un fragmento de 141 pb del gen *ompA* de *C. trachomatis* y un fragmento de 166 del gen *girasa A* de *Neisseria gonorrhoeae*; la detección de los fragmentos resultantes se hace tras la hidrólisis de una sonda marcada con rodamina 6G. En este estudio solo se hizo la amplificación para *C. trachomatis.*

### Tipificación del HPV

La detección y genotipificación del HPV se hicieron en todas las muestras de las participantes tal como se describe en el estudio de Sánchez, *et al.*^11^: se hizo una prueba de PCR con los iniciadores Gp5+/Gp6+ que amplifican un fragmento de 150 pb del gen *L1* conservado en todos los genotipos. Dicho fragmento se hibridó con sondas específicas para 37 genotipos del virus HPV ^14^.

### Análisis de datos

Las variables cualitativas se describieron usando porcentajes y, las cuantitativas, con medias y medidas de dispersión (desviación estándar). Se hicieron comparaciones con las pruebas de Mann-Whitney y de ji al cuadrado para evaluar la asociación bivariada entre las variables independientes y la infección con *C. trachomatis*. Además, se evaluó esta asociación utilizando razones de prevalencia (RP) ajustadas por edad con sus respectivos intervalos de confianza del 95 % (IC_95%_). Las razones de prevalencia se estimaron usando modelos de regresión binomial, así como modelos multivariados para estudiar la asociación conjunta de la edad, uso de anticonceptivos hormonales, embarazo, e infección por HPV++ (cualquier genotipo), consumo de cigarrillo y resultado de la citología. Para este análisis, se empleó la librería GLM del paquete estadístico R (R Core Team, 2013). La concordancia entre las pruebas diagnósticas se determinó y evaluó mediante el cálculo del índice kappa.

### Consideraciones éticas

El estudio fue aprobado por los comités de ética de la Facultad de Medicina y de la Sede de Investigación Universitaria de la Universidad de Antioquia.

## Resultados

De las 1.282 mujeres, se incluyeron finalmente 1.087. Las razones de exclusión de las 195 participantes restantes fueron las siguientes: 32 muestras correspondían a mujeres de menos de 20 años; 136 fueron negativas para β-globina, y en 27 el ADN era insuficiente.

La edad media (± desviación estándar) de las participantes fue de 39,4 (±12,5) años, la mayoría en el rango de 26 a 35 años. La edad media de inicio de relaciones sexuales fue de 19,3 ± 4,2 años. El 38,5 % de las participantes que entregaron dicha información reportó un solo compañero sexual durante toda la vida. El 80,5 % había estado en embarazo alguna vez, el 87,0 % reportó usar anticonceptivos hormonales y el 61,7 % usaba condón siempre o algunas veces. El 65,8 % reportó no haber fumado cigarrillos nunca y el 91,9 % no reportó antecedentes de infección de transmisión sexual. Las demás características demográficas y variables asociadas con conducta sexual, salud reproductiva y consumo de cigarrillo, se resumen en el cuadro 1.

**Cuadro 1:** Descripción de la población de studio

Variable (media/DE)	Frecuencia absoluta	Frecuencia relativa
Edad (años) (39,4/12,5)		
22-25	160	17,3
26-35	304	32,8
36-45	293	31,6
46-55	205	22,1
≥55	125	13,5
Lugar de nacimiento		
Valle de Aburrá	600	55,2
En Antioquia, fuera del Valle de Aburrá	326	30,0
Fuera de Antioquia	146	13,4
Sin información	15	1,4
Edad de primera relación sexual (años) (19,3/4,2)		
≥20	286	26,3
16-19	347	31,9
≤15	111	10,2
Sin información	343	31,6
Uso de anticonceptivos hormonales		
Nunca	211	19,4
Alguna vez	869	79,9
Sin información	7	0,6
Edad primer embarazo (años) (22,6/5,3)		
Sin información	548	50,4
Uso de condón		
Nunca	159	14,6
Ocasionalmente	182	16,7
Siempre	74	6,8
Sin información	672	61,8
Antecedentes de infección de transmisión sexual		
No	419	38,5
Sí	30	2,8
No sabe	7	0,6
Sin información	631	58,0
Infección por HPV++		
Negativa	973	89,5
Positiva	114	10,5
Sin información	0	0
Infección por HPV-BR		
Negativa	1036	95.3
Positiva	51	4.7
Sin información	0	0
Infección por HPV -AR		
Negativa	1024	94,2
Positiva	63	5,8
Sin información	0	0
Consumo de cigarrillo		
Nunca	713	65,6
Alguna vez	370	34,0
Sin información	4	0,4
Resultado de citología		
Negativo para malignidad	1046	96,2
ASC-US	18	1,7
LIE-BG	4	0,4
Sin información	19	1,7

DE: desviación estándar; HPV++: infección por cualquier genotipo; HPV-BR: infección por virus del papiloma humano genotipo de bajo riesgo; HPV-AR: infección por virus del papiloma humano genotipo de alto riesgo; ASC-US: células escamosas atípicas de significado indeterminado; LIE-BG: lesión intraepitelial de bajo grado

Con la PCR convencional, se encontró una positividad de *C. trachomatis* en 45 de 1.087 muestras (4,1 %, IC_95%_ 2,9-5,3). Con el sistema LightMix 480 HT^™^, se procesaron 135 muestras: 45 fueron positivas y 90 negativas para la PCR convencional; 32 de las 45 muestras positivas por PCR también lo fueron con el sistema LightMix 480 HT^™^, y dos de las 90 muestras negativas en la PCR fueron positivas con el sistema LightMix 480 HT^™^. La concordancia entre las dos pruebas fue del 88,0 % y el índice kappa fue de 0,7 (p<0,001), lo que indica un buen nivel de correspondencia entre ambas pruebas. Trece de las muestras positivas por PCR convencional pero negativas con el sistema LightMix 480 HT^™^ se evaluaron nuevamente con PCR convencional y se obtuvo nuevamente un resultado positivo, por lo cual quedaron definidas como muestras positivas.

Al analizar los datos de prevalencia en los diferentes rangos de edad, se observó una tendencia a la disminución de la prevalencia de *C. trachomatis* en mujeres de mayor edad. La prevalencia más alta se observó entre los 20 y 25 años (6,9 %) y, la más baja, en las mujeres de 55 años o más (1,6 %) (p=0,032) (cuadro 2).

**Cuadro 2:** Análisis univariado y multivariado para determinar la asociación de las variables demográficas con la prevalencia de *Chlamydia trachomatis*

Característica	CT+		CT-		Análisis univariado			Análisis multivariado		
	n	%	n	%	RP	IC_95%_	p	RP	IC_95%_	p
Total	45	4,1	1042	95,9						
Edad (años) (media/DE)	(36,2/12,1)		(39,5/12,6)				0,080			
20-25	11	6,9	149	93,1	1			1		
26-35	13	4,3	291	95,7	0,62	0,28-1,36	0,243	0,72	0,31-1,66	0,318
36-45	12	4,1	281	95,9	0,6	0,27-1,34		0,8	0,34-1,92	
46-55	7	3,4	198	96,6	0,5	0,20-1,26		0,69	0,26-1,86	
≥55	2	1,6	123	98,4	0,23	0,05-1,02		0,35	0,07-1,66	
Sin información	0		0		p-t 0,032			p-t 0,272		
Lugar de nacimiento										
Valle de Aburrá	29	4,8	571	95,2	1					
Resto de Antioquia	14	4,3	312	95,7	1	0,53-1,87	0,106			
Fuera de Antioquia	2	1,4	144	98,6	0,3	0,07-1,25				
Sin información	0		15							

DE: desviación estándar; CT+: pacientes diagnosticadas como positivas para *Chlamydia trachomatis*; CT-: pacientes diagnosticadas como negativas para *Chlamydia trachomatis*; IC95%: intervalo de confianza del 95 %; p-t, valor de p de tendencia

La asociación de la prevalencia de *C. trachomatis* con las variables explicativas se presenta en los cuadros 2, 3 y 4. En el análisis multivariado no se encontró asociación con las variables de conducta sexual (edad de la primera relación sexual, número de compañeros sexuales, edad del primer embarazo y uso de condón), los factores infecciosos (antecedentes de infección de transmisión sexual, infección por cualquier genotipo de HPV (HPV++), la infección por un genotipo de HPV de alto riesgo (HPV-AR), la infección por un genotipo de HPV de bajo riesgo (HPV-BR), el resultado de la citología ni el consumo de cigarrillo.

**Cuadro 3:** Análisis univariado y multivariado para determinar la asociación de las variables de conducta sexual y reproductiva y de consumo de cigarrillos con la prevalencia de *Chlamydia trachomatis*

Característica	CT+		CT-		Análisis univariado			Análisis multivariado		
	n	%	n	%	RP	IC_95%_	p	RP	IC_95%_	p
Edad de primera relacion sexual	(18,8/3,3)		(19,3/4,2)							
(anos) (media/DE)										
≥20	14	4,9	272	95,1	1					
16-19	10	2,9	337	97,1	0,49	0,21-1,14	0,313			
<15	6	5,4	105	94,6	0,84	0,32-2,24				
Sin informacion	15		328		p-t 0,798					
Parejas sexuales (media/DE)	(2,4/2,6)									
1	11	3,7	283	96,3	1					
2-3	17	5,2	310	94,8	1,27	0,59-2,73	0,108			
4+	2	1,4	140	98,6	0,35	0,08-1,58				
Sin información	15		309		p-t 0,439					
Uso de anticonceptivos hormonales										
Nunca	10	7,1	130	92,9	1			1		
Alguna vez	34	3,6	899	96,4	0,46	0,23-0,91	0,072	0,46	0,23-0,91	0,034
Sin información	1		13							
Embarazo										
Nunca	14	6,6	197	93,4	1			1		
Alguna vez	30	3,5	839	96,5	0,56	0,29-1,09	0,048	0,55	0,28-1,07	0,121
Sin informacion	1		6							
Edad primer embarazo (anos)	(22,2/4,8)		(22,6/5,3)		1	0,92-1,08	0,725			
(media/DE)										
Sin información	24		524							
Uso de condon										
Nunca	6	3,8	153	96,2	1	0,24-2,53	0,499			
A veces	7	3,8	175	96,2	0,78	0,03-2,47				
Siempre	1	1,4	73	96,2	0,28					
Sin informacion	31		641		p-t 0,416					
Consumo de cigarrillo										
Nunca	39	5,5	674	94,5	1			1		
Alguna vez	6	1,6	364	98,4	0,3	0,13-0,71	0,001	0,34	0,15-0,81	0,006

DE: desviación estándar; CT+: pacientes diagnosticadas como positivas para *Chlamydia trachomatis*; CT-: pacientes diagnosticadas como negativas para *Chlamydia trachomatis*; IC95%: intervalo de confianza del 95 %; p-t: valor de p de tendencia

Aunque algunas diferencias no fueron estadísticamente significativas, se observó una prevalencia mayor (5,2 %) en las mujeres que habían tenido entre dos y tres compañeros sexuales durante la vida que en quienes habían tenido solo un compañero (RP=1,27, IC_95%_ 0,59-2,73) (cuadro 3). Aunque la variable de uso de condón no se evaluó en todas las mujeres, en el grupo en el que sí se hizo (n=415) la prevalencia de *C. trachomatis* fue menor (1,4 %) en aquellas que reportaron usar condón siempre comparadas con quienes reportaron no haberlo usado nunca (RP=0,28, IC_95%_ 0,03-2,47) (cuadro 3). Ninguna mujer positiva para *C. trachomatis* reportó haber tenido antecedentes de infección de transmisión sexual (cuadro 4).

**Cuadro 4:** Análisis unvariado y multivariado para determinar la asociación entre las variables de los factores infecciosos, los resultados de la citología y el consumo de cigarrillo con la prevalencia de Chlamydia trachomatis

Característica	CT+		CT-		Análisis univariado			Análisis multivariado		
	n	%	n	%	RP	IC_95%_	p	RP	IC_95%_	p
Antecedentes de ITS										
No	15	3,6	404	96,4	1					
Si	0	0,0	30	100	0	0,00-0,00	0,275			
No sabe	0	0,0	7	100	0	0,00-0,00				
Sin información	30		601							
Infección por HPV++								1		
Negativa	39	4,0	934	96	1	0,56-3,02	0,539	1,03	0,44-2,41	0,626
Positiva	6	5,3	108	94,7	1,3					
Sin información	0		0							
Infección por HPV-BR										
Negativa	43	4,2	993	95,8	1					
Positiva	2	3,9	49	96,1	0,91	0,23-3,66	0,936			
Sin información	0		0							
Infección por HPV-AR										
Negativa	41	4,0	983	96	1					
Positiva	4	6,3	59	93,7	1,59	0,59-4,32	0,397			
Sin información	0		0							
Consumo de cigarrillo										
Nunca	39	5,5	674	94,5	1			1		
Alguna vez	6	1,6	364	98,4	0,3	0,13-0,71	0,001	0,34	0,15-0,81	0,006
Sin información	0		4							
Resultado de citología										
Negativo para malignidad	43	4,1	1003	95,9	1			1		
ASC-US	1	5,6	17	94,4	1,18	0,17-8,21	0,334	1,3	0,18-9,47	0,313
LIE-BG	1	25,0	3	75	5,52	1,04-29,2		6,87	1,23-38,2	
Sin información	0		19		p-t 0,176			p-t 0,071		

CT+: pacientes diagnosticadas como positivas para *Chlamydia trachomatis*; CT-: pacientes diagnosticadas como negativas para *Chlamydia trachomatis*; p-t: valor de p de tendencia; ITS: infección de transmisión sexual; HVV++: infección por virus del papiloma humano de cualquier genotipo; HPV-BR: infección por virus del papiloma humano de genotipo de bajo riesgo; HPV-AR: infección por virus de papiloma humano de genotipo de alto riesgo; ASC-US: células escamosas atípicas de significado indeterminado; LIE-BG: lesión intraepitelial de bajo riesgo

En cuanto al embarazo, el 80,5 % de las mujeres reportó que, por lo menos, una vez había estado en embarazo y, al ajustar dicha comparación por la edad, se encontró que la prevalencia de *C. trachomatis* fue significativamente mayor (6,6 %) en las mujeres que nunca habían estado en embarazo comparadas con las que sí habían estado (RP=0,56, IC_95%_ 0,29-1,09; p=0,048). Sin embargo, cuando se hizo el análisis multivariado, la significación se perdió (p ajustado=0,121) (cuadro 3).

El 87 % de las participantes habían usado anticonceptivos hormonales alguna vez durante su vida. Se encontró una prevalencia significativamente menor de la infección (7,1 %) en mujeres que nunca habían usado anticonceptivos hormonales. En el análisis multivariado, esta diferencia se mantuvo (p ajustado= 0,034) (cuadro 3).

Al evaluar la relación con la infección por HPV*,* se encontró que la prevalencia de *C. trachomatis* fue mayor en las mujeres con infección HPV++ (RP=1,30, IC_95%_ 0,56-3,02), pero no fue estadísticamente significativa (cuadro 4). Al discriminar por tipo de HPV (alto riesgo-AR o bajo riesgo-BR), la prevalencia de *C. trachomatis* fue mayor (6,3 %) en las mujeres infectadas con HPV-AR que en las infectadas con HPV-BR (3,9 %); no obstante, estas dos comparaciones tampoco tuvieron significación estadística (p=0,397 y 0,936) (cuadro 4). La prevalencia de *C. trachomatis* en las mujeres con lesión intraepitelial de bajo grado (LIE-BG) fue seis veces mayor (25,0 %) que en aquellas con resultado citológico negativo para neoplasia maligna; sin embargo, debido a la poca frecuencia de este resultado, la diferencia no fue estadísticamente significativa (cuadro 4).

El 65,8 % de las mujeres no reportó haber consumido cigarrillo. Después de ajustar por la edad, se encontró que la prevalencia de *C. trachomatis* fue menor en las mujeres que alguna vez habían fumado (1,6 %; RP=0,30; IC_95%_ 0,13-0,61), diferencia con significación estadística tanto en el análisis univariado como en el muiltivariado (p=0,001 y 0,006) (cuadro 4).

## Discusión

La infección por *C. trachomatis* se ha estudiado poco en la población colombiana a pesar de que puede producir graves complicaciones, y Medellín no es una excepción. El único estudio sobre la infección se llevó a cabo hace 32 años ^7^. En las últimas décadas, no solo ha habido cambios en la conducta sexual de la población, sino que, además, hoy se cuenta con métodos moleculares más sensibles y específicos, que, a diferencia de la serología, pueden medir de una manera exacta la exposición actual a esta infección.

La prevalencia encontrada en este estudio (4,1 %) es similar a la reportada en Bogotá en mujeres asintomáticas (2,24-5,0 %) y en otros países como Argentina (5 %), Estados Unidos (4,2 %), España (4,0 %), Kenia (6,0 %) y Suecia (5,6 %), lo que indica que la infección está desatendida en nuestro medio tanto como en las diferentes zonas geográficas ^3^^-^^5^^,^^10^^,^^15^^-^^19^.

Si bien en este estudio el 83 % de las participantes tenía una edad superior a los 25 años, se encontró una tendencia a la disminución de la infección por *C. trachomatis* a medida que aumentaba la edad, situación también observada en otros lugares del mundo, por lo que se considera que la infección por *C. trachomatis* es dependiente de la edad, siendo más común en mujeres sexualmente activas menores de 25 años ^20^^,^^21^. La tendencia a la disminución de la infección a medida que aumenta la edad podría explicarse por una reducción del número de nuevas parejas sexuales, la presencia de factores biológicos protectores para el desarrollo de la infección y promotores de una rápida resolución ^2^.

Aunque no encontramos relación entre los antecedentes de otras infección de transmisión sexual y el riesgo de infección, es de anotar que ninguna de las mujeres positivas para *C. trachomatis* reportó tener antecedentes de infección de transmisión sexual, lo que pone de relieve el carácter silencioso de esta infección que lleva a que una gran proporción de las infectadas no sean conscientes de estarlo ^22^*.* Sin embargo, cabe resaltar que en el caso de este estudio fue difícil medir la variable de antecedentes de infección de transmisión sexual y solo se pudo obtener información en una porción muy reducida de la población muestreada.

Se encontró una menor prevalencia de la infección en las mujeres que nunca habían usado anticonceptivos hormonales. En los estudios consultados dicha asociación no es muy consistente y es muy difícil comparar nuestros resultados con los de estos, ya que, en la mayoría de ellos, solo se hace referencia a los anticonceptivos orales ^23^. En el estudio de Molano, *et al.*, en población colombiana no se encontró ningún tipo de asociación con el uso de anticonceptivos en general ^24^.

Fue interesante encontrar una menor prevalencia de *C. trachomatis* en las mujeres que habían consumido cigarrillo en comparación con las que no (p ajustado=0,006), lo que contradice lo reportado por Haar, *et al.*^25^. Esto puede deberse a causas atribuibles a otras variables, o a sesgos por una posible falsa información de las participantes en el momento de responder la encuesta, o a otras variables de confusión, como el comportamiento sexual de sus compañeros; infortunadamente, en el presente estudio no se contaba con dicha información.

En concordancia con otros estudios, no se encontró una clara asociación entre la infección por *C. trachomatis* y la infección por el HPV ^26^^-^^28^ Neisseria gonorrhoeae, Treponema pallidum, herpes simplex virus type II (HSV-2. Sin embargo, cabe resaltar que en el presente trabajo solo se incluyeron mujeres con citología normal o lesión intraepitelial de bajo grado, por lo que la comparación con los que han analizado el papel de *C. trachomatis* en el desarrollo del cáncer cervical no es factible. En un futuro se podría detectar *C. trachomatis* en las muestras de casos de cáncer cervical para lograr una estimación adecuada de su asociación con la neoplasia cervical. Sin embargo, encontramos una mayor prevalencia en las mujeres infectadas con genotipos de alto riesgo, aunque dicha diferencia no fue estadísticamente significativa.

Los resultados obtenidos en este trabajo demuestran que la prevalencia de *C. tracomatis* en Medellín es similar a la observada en otros lugares y es mayor en mujeres jóvenes con una vida sexual más activa. Cabe anotar que, en este estudio, el nivel de significación de 0,05 y la prevalencia observada de 4,1 % para el tamaño de muestra tuvieron un poder estadístico mayor al 80 % para detectar diferencias significativas entre dos prevalencias que difirieran en más de 2,7 %. La precisión de este estudio fue de ±1,16 % que, en relación con la prevalencia observada, corresponde a una precisión relativa del 72 %. Por lo tanto, la potencia y el grado de precisión fueron adecuados para hallar diferencias significativas entre las variables demográficas, la conducta sexual y los factores infecciosos evaluados para determinar los diferentes factores de riesgo de la infección.

En conclusión, en Medellín se encontró una relación entre las variables de edad, consumo de cigarrillo, uso de anticonceptivos hormonales y resultado de la citología y la presencia de infección por *C. trachomatis*. No se encontró una asociación estadísticamente significativa con la infección por el HPV. Según estos resultados, se puede decir que en la ciudad la infección asintomática por *C. trachomatis* es un importante problema de salud pública y su frecuencia es similar a la encontrada en diferentes regiones geográficas, por lo que se necesitan medidas de tamización en nuestra población.
